# 

**Published:** 1881-10

**Authors:** 


					﻿ESTABLISHED IN 1855.
Old Series.	OCTO'R'E'R 1RR1	New Series.
Vol. XIX-No. 7.	UL1UDXA, 1OO1.	Vol. I-No. 1.
ATLANTA
MEDICAL REGISTER.
(ATLANTA MEDICAL AND SURGICAL JOURNAL.)
-----------------------
(	EDITOBS:	<--
JNO. THAD. JOHNSON, M.D., JAS. B. BAIRD, M.D.
PUBLISHED BY H. H. DICKSON, AT $2.50 A YEAR IN ADVANCE.
4
ATLANTA, GEORGIA:
U. II. DICKSON, BOOK AND JOB PRINTER AND BOOKBINDER.
1RR1.
				

